# Improving the Performance of the Layered Nickel Manganese Oxide Cathode of Sodium-Ion Batteries by Direct Coating with Sodium Niobium Oxide

**DOI:** 10.1021/acsami.4c09706

**Published:** 2024-10-09

**Authors:** Sergio Lavela, Antônio Carlos do Nascimento Santos, Fabiana Villela da Motta, Mauricio Roberto Delmonte Bomio, Pedro Lavela, Carlos Pérez Vicente, José Luis Tirado

**Affiliations:** † LSQM − Laboratory of Chemical Synthesis of Materials, Department of Materials Engineering, Federal University of Rio Grande do Norte − UFRN, P.O. Box 1524, Natal, RN, 59078-970, Brazil; ‡ Departamento de Química Inorgánica e Ingeniería Química, Instituto Químico para la Energía y el Medioambiente, Universidad de Córdoba, Edificio Marie Curie, Campus de Rabanales, 14071 Córdoba, Spain

**Keywords:** sodium-ion batteries, coating, layered oxides, NaNbO_3_, nickel, manganese oxide

## Abstract

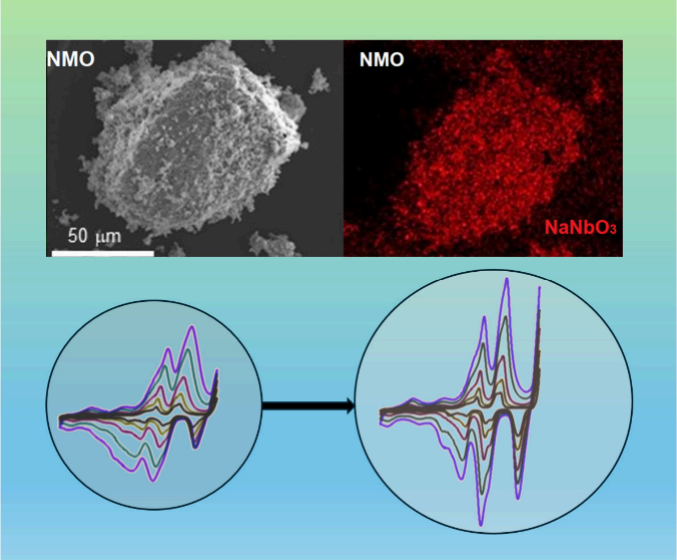

This research highlights the efficacy of NaNbO_3_ as a coating for P2-Na_2/3_Ni_1/3_Mn_2/3_O_2_ cathodes in sodium-ion batteries. The coating enhances the kinetic behavior and cyclability of the electrochemical cells, as shown by electrochemical measurements. XRD analysis indicates that Nb does not incorporate into the cathode structure, implying a physical interaction between the coating and the cathode material. XRF analysis and EDX mapping confirm the actual composition and uniform dispersion of elements throughout the sample, while the electron micrographs evidence the occurrence of NaNbO_3_ particles modifying the surface of the layered oxide. The Ni^4+^/Ni^3+^ and Ni^3+^/Ni^2+^ redox pairs, along with the partially reversible oxidation of oxide to peroxide anions, contribute significantly to cell capacity, as revealed by XPS spectra. This last effect and the appearance of a co-intercalated phase at high voltage are positive factors to provide fast kinetics. Cyclic voltammograms show that samples coated with 2–3% NaNbO_3_ have superior rate capability, with high capacitive response and apparent diffusion coefficients. These samples also have low impedance at the electrode–electrolyte interface, which helps deliver a high capacity at 5C. Further cycling at 1C shows improved cyclability in the bare and 3% coated samples, due to their higher diffusion coefficients on charging. Notably, the 3% NaNbO_3_-coated sample exhibits excellent cyclability below 0 °C, making it a promising cathode material for sodium-ion batteries.

## 1 Introduction

Early concerns regarding the commercialization of sodium-ion batteries (SIB) as competitors to lithium-ion batteries (LIB) gradually disappear.^1−5^ The inherent lesser negative reduction potential and larger size of sodium than lithium initially pointed out as a critical limitation to achieve enough performing batteries. Notwithstanding, the research on new electrode materials has achieved relevant breakthroughs that have eventually attracted the market attention to sodium-ion batteries. Especially, Prussian blue analogues, metal sulfides, metal–organic frameworks, NASICON-type phosphates, and layered metal oxides have demonstrated that their open frameworks are suitable to ensure fast ion diffusion at high working voltages to play the role of cathodes.^6−11^

Energy density is no longer the primary indicator to pursue. Sodium-ion batteries (SIBs) can be more cost-effective than lithium-ion batteries (LIBs) due to the abundance and uniform distribution of sodium in the Earth’s crust.^12−14^ Moreover, efficient procedures for extracting sodium from seawater are known. Additionally, SIBs exhibit lower tendencies to form dendrites, which enhances their stability and safety during cycling.^15^ Specifically, different reports envision SIB application in large-scale grid storage to increase the integration of renewable energy sources.^15−19^

Layered oxides with a Na_*x*_MO_2_ (where 0.67 ⩽ x ⩽ 1) nominal stoichiometry, where M represents transition elements such as Mn, Fe, Ni, etc., exhibit significantly high output voltage, large theoretical capacity, and simple synthetic routes. These characteristics make them attractive cathode materials for practical SIBs. Their typical structures belong to the O3-type and P2-type crystal families. Both types have advantages and limitations, making it challenging to determine the most suitable structure for eventual commercial use. Notably, sodium diffusivity is better in the P2-type phase, thanks to low-energy conduction pathways where Na^+^ ions, located at triangular prism sites within the interlayer space, migrate directly through rectangular faces. In contrast, Na^+^ ions transport through small triangular faces in an O3-type structure.^20^

P2-type Na_2/3_Ni_1/3_Mn_2/3_O_2_ is a promising positive electrode. Transition metals and sodium are in alternating slabs, allowing Na^+^ ions to migrate through low-energy conduction pathways created by triangular prism sites sharing faces.^20^ Its high output voltage, large capacity, moisture resilience, and ease of large-scale manufacturing make this oxide an appealing cathode material for practical sodium-ion batteries (SIBs).^21,22^ Despite its interesting features, challenging issues need to be overcome. The multiple voltage steps observed in the charge/discharge come from the phase transitions, resulting in various Na^+^/vacancy ordered patterns that form at specific Na stoichiometries. This phenomenon is commonly attributed to the strong repulsion among Na^+^ ions in the Na layer and charge ordering in the transition metal layer.^23^ It decreases the Na-ion diffusion coefficient and limits the dimensionality of Na-ion transport, harming the electrochemical properties.^24^ Also, the capacity delivered from the 4.2 V plateau decays abruptly upon cycling, accompanied by a sharp increase in polarization. The occurrence of a biphasic region involving P2–O2 transitions leads to severe structural degradation and a dramatic loss of capacity.^25^ Several strategies are proposed to optimize cell performance. Our research group has experience with metal doping,^26^ partial replacement of nickel,^27^ materials with controlled morphology,^28^ and new preparative routes.^29,30^ Alternatively, surface modification is a feasible approach to enhance the electrochemical performance of cathodes by improving cyclability at high rates, avoiding electrode degradation, and mitigating undesirable phase transitions. For instance, CeO_2_ coating stabilizes the redox reaction of lattice oxygen above 4.2 V, contributing favorably to a large and stable reversible capacity.^31^ Additionally, Al_2_O_3_ coating on Na_2/3_Ni_1/3_Mn_2/3_O_2_ suppresses unfavorable high voltage side reactions and prevents metal oxide layer exfoliation, resulting in enhanced cycling performance.^32^

These results prompted us to seek an effective novel surface modifier to improve the electrochemical behavior of this layered oxide. In this way, sodium niobate (NaNbO_3_), with a perovskite-type structure, features interesting properties in applications such as piezoelectricity, ferroelectricity, and photocatalysis.^33,34^ Although its open structure and rich redox chemistry (Nb^5+^/Nb^4+^/Nb^3+^) would favor high specific capacity as an anode, its poor electrical conductivity has limited its applicability.^35,36^ Recently, Zhou et al. reported that attempts to dope a layered oxide with Nb resulted in an unexpected surface reorganization of an atomic-scale NaNbO_3_ coating layer that effectively prevented the dissolution of metals and surface side reactions.^37^

This study aims to evaluate, for the first time, the effect of NaNbO_3_ coating on P2–Na_2/3_Ni_1/3_Mn_2/3_O_2_. Several samples with varying contents of the coating agent were prepared, and their effects on textural and structural properties will be examined using X-ray diffraction, electron microscopy, and X-ray photoelectron spectroscopy. Sodium half-cells, assembled with the studied material as working electrodes using both cyclic voltammetry and galvanostatic techniques are used to unveil the reliability of the optimized material as a cathode for Na-ion cells.

## 2 Materials and Methods

### Synthesis

A sample with P2-Na_2/3_Ni_1/3_Mn_2/3_O_2_ (NMO@Nb0) nominal composition was obtained by a sol–gel procedure. The sol–gel synthesis resided on the use of citric acid (Synth, 99%) as a chelating agent in a 3 to 2, citric to metal ratio. Thus, stoichiometric amounts of Ni(NO_3_)_2_.6H_2_O (Sigma-Aldrich, 99%), Mn(NO_3_)_2_.4H_2_O (Alfa Aesar, 98%), were dissolved in 100 mL containing the citric acid solution (solution A). Finally, sodium acetate with 5% excess (Vetec, 99%), was dissolved in 50 mL of distilled water (Solution B). Solution B was slowly dropwise into solution A, until complete mixing of the solutions. The solution was evaporated at 120 °C and the resulting gel calcinated at 450 °C for 3h. The obtained materials were uniaxially pressed into pellets and annealed in an alumina crucible at 900 °C with a 2 °C min^–1^ heating rate for 12h.

The NaNbO_3_ resin was synthesized by the Pechini method. The polymeric resin was produced using 17.7 g of citric acid (Synth 98%), used as a chelating agent, and 11.8 g of ethylene glycol (Synth) as a polymerizing reagent. The precursors of the NaNbO_3_ phase were 5.97g niobium ammonium oxalate (NH_4_H_2_NbO(C_2_O_4_)_2_.3H_2_O), provided by CBMM (Brazilian Metallurgy and Mining Company), and 1.29g of sodium nitrate (Synth 98%). Finally, the viscosity of the resin was adjusted to 22 cP using a Brookfield viscometer and subjected to a gravimetric test to determine the amount of NaNbO_3_/ml of resin. In a second step, 500 mg of Na_0.67_Ni_0.33_Mn_0.67_ were coated by a chemical route involving the addition of 1%, 2%, and 3% of NaNbO_3_ resin in 50 mL of ethanol (Synth 99% v/v) using a pulse-ultrasonic probe (Ultronique) immersed in the precursor solution for 30 min. The stirring conditions were set at 750 W 20 kHz and a pulse duration of 10 s. Afterward, the resulting material was further dried at 120 °C overnight, pestled in an agate mortar, and annealed at 800 °C with a 5 °C min^–1^ heating rate for 2 h. These samples will be labeled as NMO@Nb(%) as follows: NMO@Nb1, NMO@Nb2, and NMO@Nb3.

### Structural and Morphological Characterization

X-ray diffraction patterns were scanned between 10 and 80° (2-theta) at 1°/min by using an XRD-6000 Shimadzu diffractometer furnished with Cu Ka radiation and a graphite monochromator. TOPAS software was useful to determine the unit cell parameters. Field Emission-Scanning Electron micrographs (FE-SEM) and EDX maps were recorded in a JSM-7800F Prime JEOL Microscope. High-Resolution Transmission Electron Microscopy (HRTEM) images were recorded in a TALOS F200i. The elemental composition was determined using a Rigaku ZSX Primus IV sequential wavelength dispersive X-ray fluorescence (XRF) spectrometer.

### Electrochemical Characterization

Swagelok-type sodium half-cells were composed of working electrodes (approximately 3 mg/cm^2^) consisting of the following components: Active material (70%), Acetylene black (20%), and polyvinylidene fluoride (PVDF) (10%), mixed in *N*-methyl-2-pyrrolidone (Emplura, 99.5%) and gently stirred for at least 2 h. The resulting homogeneous paste was spread onto aluminum disks (diameter = 9 mm) and then vacuum-dried at 120 °C for several hours. The counter electrode was metallic sodium (Panreac, 99.8%). The electrolyte solution consisted of 1 M NaPF_6_ (Strem, > 99%) in propylene carbonate (PC) (Sigma-Aldrich, 99.7%) with 2% wt. FEC (fluoroethylene carbonate) (Sigma-Aldrich, 99%). This solution impregnated glass fiber disks (GF/A-Whatman) separators. The cells were assembled in an argon-filled MBraun glovebox, where O_2_ and H_2_O traces could be monitored. Galvanostat/Potentiostat multichannel systems (Biologic) monitored the electrochemical tests. The rate capability was determined by galvanostatic cycling from C/10 to 5C between 2.0 and 4.3 V versus Na^+^/Na. Cyclic voltammograms (CV) were scanned within the same potential window at rates ranging from 0.05 to 0.6 mV s^–1^. To unveil the cell’s internal impedance, Nyquist plots were recorded using Electrochemical Impedance Spectroscopy (EIS) on an SP-150 Biologic. The open circuit voltage was perturbed with an oscillating signal (amplitude = 5 mV) from 100 kHz to 2 mHz. Cells that underwent one cycle at C/10 were allowed to relax for at least 12 h to reach a quasi-equilibrium state.

## 3 Results and Discussion

**Figure 1:**
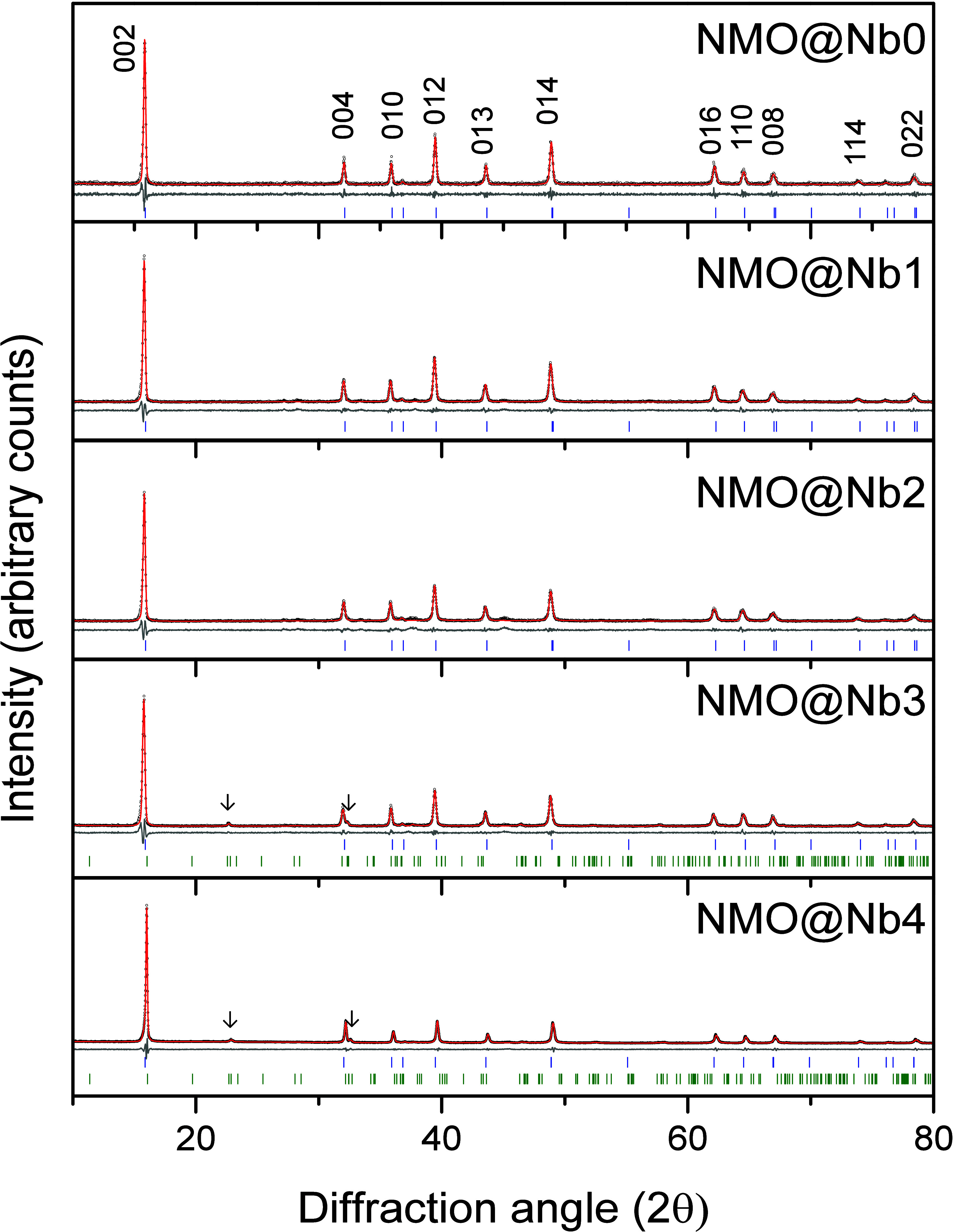
X-ray diffraction patterns of bare and NaNbO_3_-coated P2-Na_2/3_Ni_1/3_Mn_2/3_O_2_ samples (black dots). Calculated patterns (red) and differential curves (gray) are included for each sample. DIF patterns for P2-Na_2/3_Ni_1/3_Mn_2/3_O_2_ (blue) and NaNbO_3_ (green) are depicted. Arrows indicate the most intense reflection appearing for NaNbO_3_. Miller indices of P2-Na_2/3_Ni_1/3_Mn_2/3_O_2_ are shown for NMO@Nb0.

Figure S1 (Supporting Information) shows the XRD pattern of the orthorhombic NaNbO_3_ coating agent. The diagram was indexed in the *Pbcm* space group of the orthorhombic system, revealing its purity and crystallinity, along with cell parameters of a = 5.517(2) Å, b = 5.568(2) Å, and c = 15.533(4) Å. The diagrams of bare and coated samples feature a set of narrow reflections, indexed in the *P*6_3_/*mmc* space group of the hexagonal system, corresponding to the structure of the P2–Na_2/3_Ni_1/3_Mn_2/3_O_2_ material used as an electrochemically active substrate (Figure 1). This material exhibits an open framework, consisting of alternate slabs of (Mn, Ni)O_6_ octahedra and NaO_6_ prisms sharing faces, which facilitates the diffusion of sodium ions.^38,39^ Their lattice parameters, collected in Table 1, are like those reported in the literature.^40^ As the coating level increases, these values remain unvaried, suggesting that the incorporation of Nb into the structure of the substrate cannot be inferred. As mentioned above, Zhou et al. reported that Nb doping in Na_0.67_Li_0.1_Fe_0.5_Mn_0.38_Nb_0.02_O_2_ led to the in situ formation of a protective NaNbO_3_ film, due to the more negative formation energies of Nb^5+^ and Na^+^ in the surface as compared with the bulk.^37^ Our results indicate that the opposite effect involving the incorporation of Nb into the framework departing from a depositing procedure is not favored. The presence of the NaNbO_3_ phase is hardly visible due to the low contents applied. Anyway, the most intense reflection could be observed for NMO@Nb4 and was remarked by arrows in Figure 1. Table 2 shows the elemental composition of the Na_2/3_Ni_1/3_Mn_2_/_3_O_2_ substrate and NaNbO_3_ content deposited onto the coated samples, determined by X-ray fluorescence. These values show a close similarity to the theoretical ones, evidencing the reliability of the synthetic procedure to obtain the materials with the expected stoichiometry.

**Table 1:** Unit Cell Parameters, Indexed in the *P*6_3_/*mmc* Space Group, for the P2-Na_2/3_Ni_1/3_Mn_2/3_O_2_ Samples

Sample	*a*, Å	*c*, Å	Volume, Å^3^
NMO@Nb0	2.882(2)	11.136(6)	80.09(1)
NMO@Nb1	2.883(1)	11.135(4)	80.16(6)
NMO@Nb2	2.883(2)	11.135(7)	80.1(1)
NMO@Nb3	2.882(1)	11.136(6)	80.1(1)
NMO@Nb4	2.882(1)	11.136(1)	80.1(1)

**Table 2:** Na, Mn, and Ni Contents from the Stoichiometric Ratio and Data Determined by XRF

		Mass percentage/%			
Sample		Na	Mn	Ni	% NaNbO_3_
NMO@Nb0	Calculated	15.4	36.8	19.4	
	Experimental	14.2	38.6	18.8	
NMO@Nb1					1.11
NMO@Nb2					1.99
NMO@Nb3					2.91
NMO@Nb4					3.88

**Figure 2:**
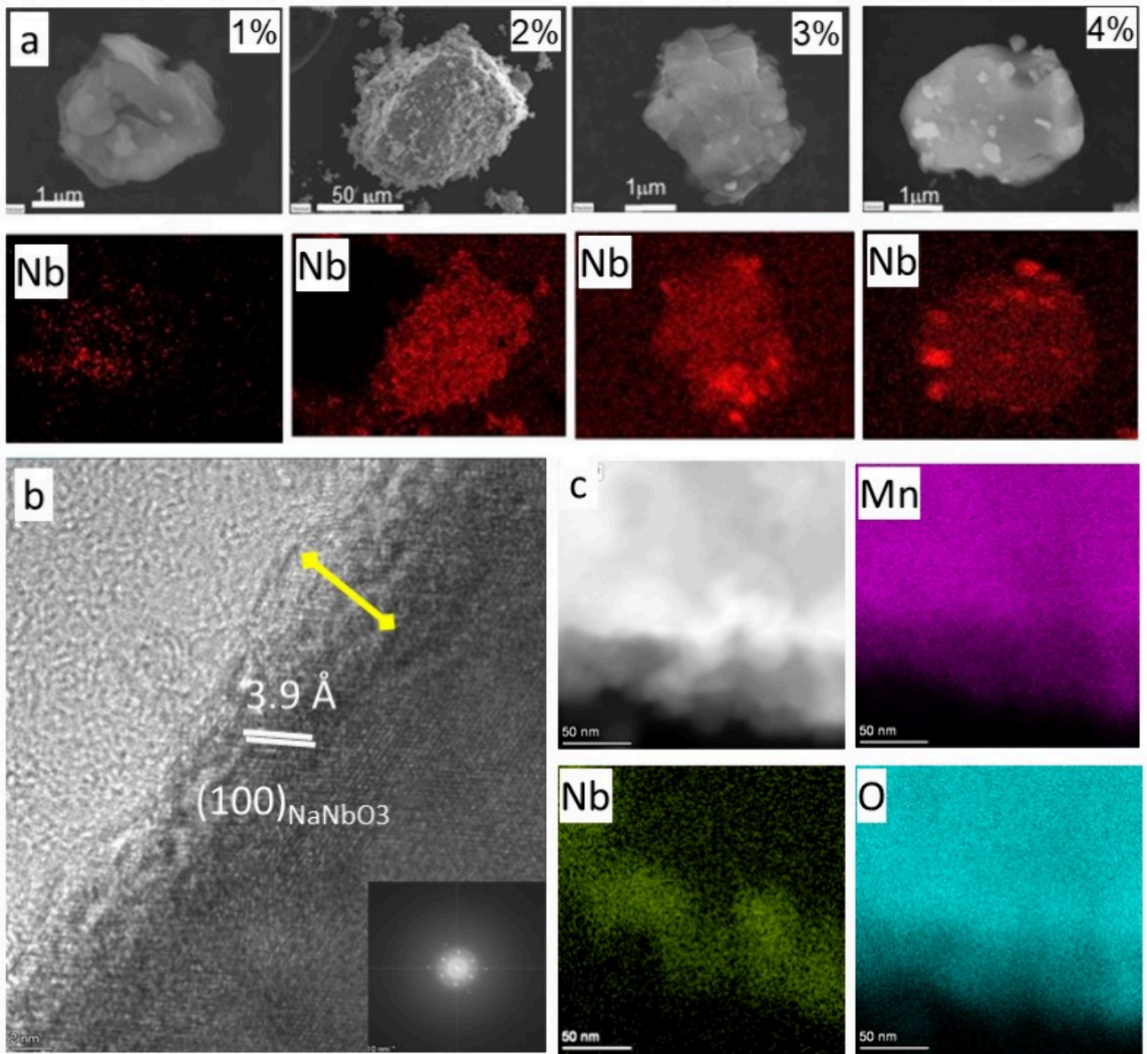
a) FE-SEM micrographs and Nb EDX maps of NaNbO_3_-coated samples; b) HRTEM image with the inset showing the FFT of the bulk image corresponding to the ⟨001⟩ zone axis of NMO, and c) EDX maps of a surface region for NMO@Nb3.

FE-SEM images allowed us to unveil the morphology of the NaNbO_3_ deposition (Figure 2a). On increasing the NaNbO_3_ content, the Nb signal becomes more evident through the entire particle surface. Otherwise, the appearance of larger Nb-containing particles is observed for NMO@Nb3 and NMO@Nb4. Lin et al. found that the effect of Nb doping on the layered structure of a related oxide is limited, predicting that it will be detrimental whether the concentration continues to increase.^41^ Figure S2 (Supporting Information) shows the EDX spectra and elemental maps of every element for the studied coated samples. Unfortunately, this technique did not provide clear evidence of the formation of a NaNbO_3_ coating layer. In Figure 2b, high-resolution transmission electron microscopy (HRTEM) images reveal distinct textures at the particle edges in a selected coated sample. Elemental analysis conducted in these regions indicates a concentrated accumulation of niobium (Nb) in a narrow zone near the surface. Meanwhile, other representative substrate elements extend throughout the entire particle (as shown in Figure 2c).

**Figure 3:**
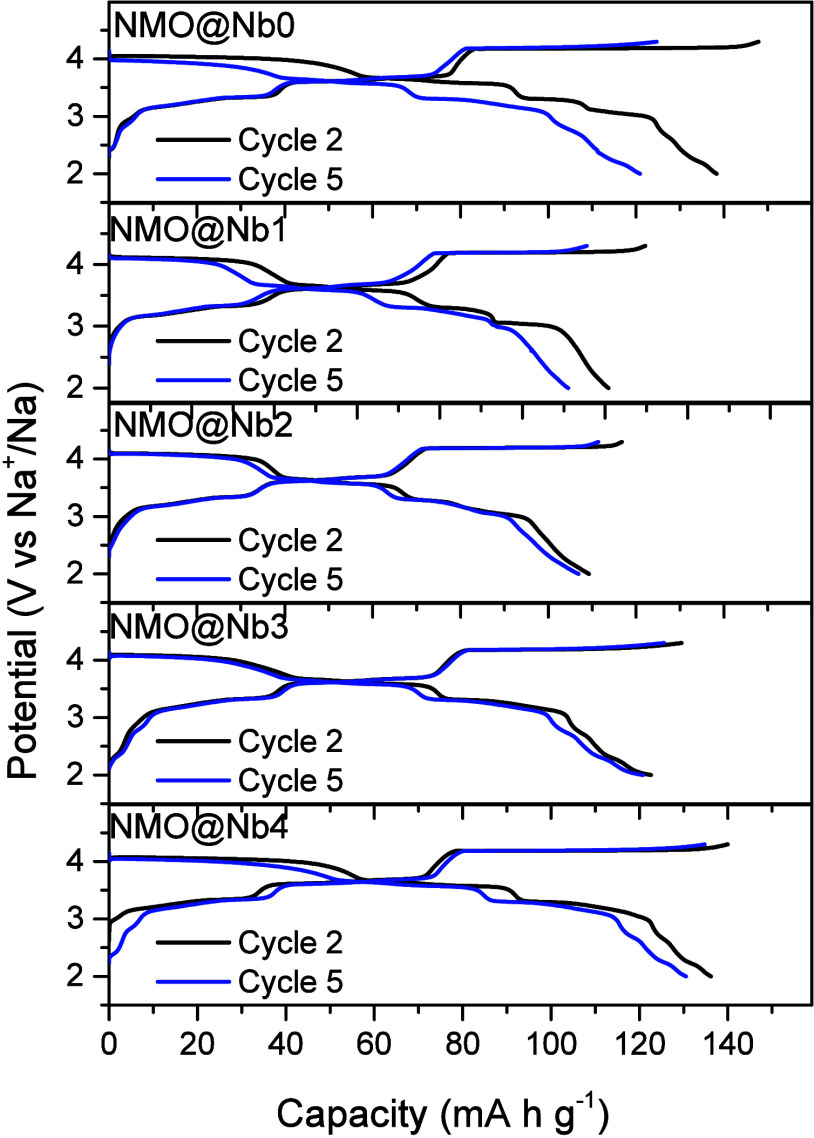
Second and fifth galvanostatic cycles for bare and NaNbO_3_-coated P2-Na_2/3_Ni_1/3_Mn_2/3_O_2_ samples recorded at C/10 between 2.0 and 4.3 V vs Na^+^/Na.

Sodium half-cells assembled with the studied samples were subjected to galvanostatic cycling. Figure 3 shows the second and fifth cycles at C/10. The overall profiles feature charge and discharge plateaus between 2.8 and 3.9 V, which are commonly attributed to the Ni^4+^/Ni^2+^ redox couple and Na-vacancy rearrangement.^42,43^ This region is responsible for the largest contribution to the cell capacity. Additionally, minor bands below 3 V resulted from a limited contribution of the Mn^4+^/Mn^3+^ couple to the overall capacity.^44^ Their limited contribution indicates an effective suppression of the deleterious Jahn–Teller effect, which occurs when a large content of trivalent manganese is present during reduction.^45^ Furthermore, reversible plateaus within the range of 3.9 to 4.3 V are observable. They may correspond to the reversible oxidation of oxide anions to peroxo species.^46−48^ To our knowledge, the use of NaNbO_3_ as cathode material for sodium-ion batteries is yet unreported. In contrast, we could find in the literature a few reports about its applicability as anode in lithium-ion batteries.^35,49^ In these reports, the faradic contributions corresponding to the reduction of Nb^5+^ occur beneath 1.5 V, out of the voltage window chosen for this study. To verify the lack of electroactivity of the coating agent in our measurement conditions, a sodium half-cell, assembled with pure NaNbO_3_, was cycled between 1.5 and 4.5 V at a rate of C/10 (Figure S3; Supporting Information). Notably, the recorded discharge capacity values below 7 mA h g^–1^ were a negligible percentage contribution to overall capacity. Otherwise, coated samples provided lower initial capacities, most probably due to the electrochemical inactivity of the coated agent. Despite this fact, NaNbO_3_ exerted a beneficial effect by providing a better capacity for the next cycles in coated samples.

**Figure 4:**
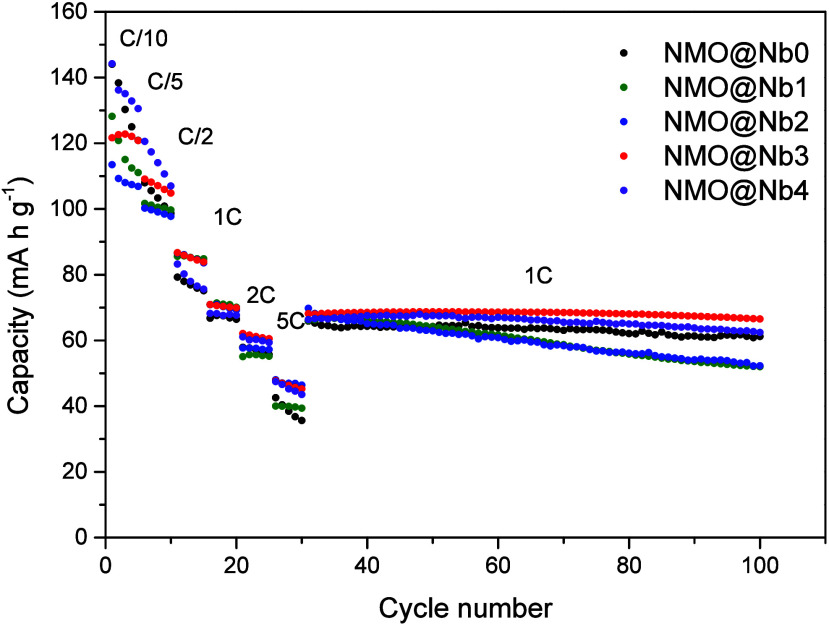
Rate capability of sodium half-cells cycled between 2.0 and 4.3 V at several rates from C/10 to 5C and then 1C.

The better reversibility of the coated samples was also exhibited at higher rates. Figure 4 plots their rate capabilities from C/10 to 5C. The initial discharge capacity value for the bare sample was 144 mA h g^–1^. It was 83% of the theoretical capacity in the assumption that all the 0.67 Na atoms per formula could be efficiently inserted. The effect of the deposition of NaNbO_3_ on the first five cycles at C/10 was a decrease in the initial capacity (122 mA h g^–1^ for NMO@Nb3). However, the capacity retention after the first cycle progressively improved by increasing the level of deposition. Thus, the capacity of the bare sample diminished to 120.9 mA h g^–1^, while that of NMO@Nb3 remained at 120.8 mA h g^–1^. It had a notorious effect on the rate capability (Figure 4). On increasing the C rate, it appears that NMO@Nb2 and NMO@Nb3 can sustain the highest capacity values at 5C 46.4 and 45.2 mA h g^–1^, respectively, while values lower than 40 mA h g^–1^ were observed for NMO@Nb0 and NMO@Nb1. These values are in correspondence to those reported for related oxides (Table S2; Supporting Information). Figure 4 also collects the capacity values for further cycling at 1C. It revealed that the surface modification with 3% NaNbO_3_ presents optimal capacity retention and the highest discharge capacity, overcoming the performance of the bare sample. As can be seen, the optimization of the percentage of coating agent is crucial.

**Figure 5:**
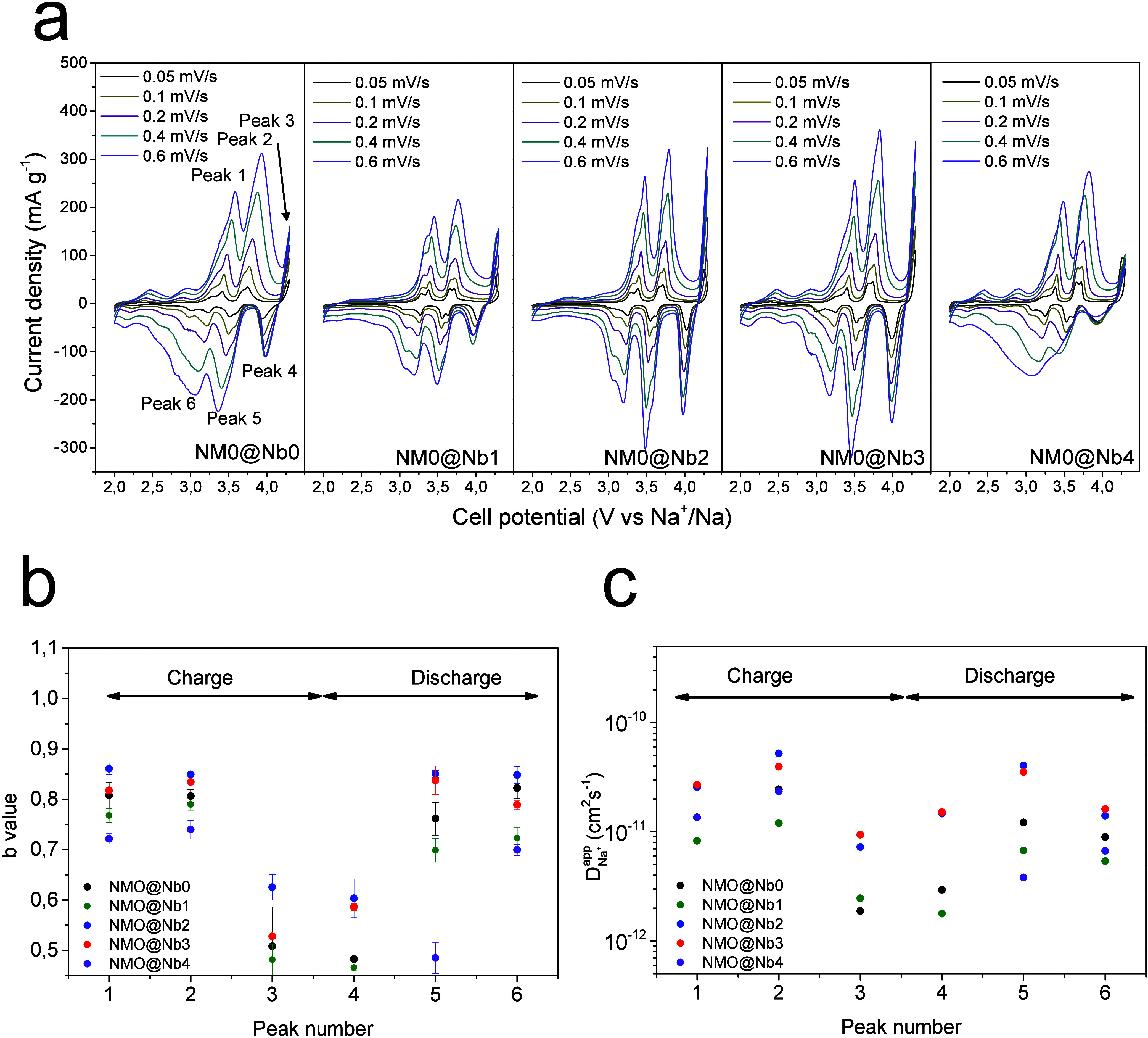
a) Cyclic voltammograms recorded at several rates from 0.05 to 0.06 mV s^–1^; the peaks used for further calculations are labeled. Plots of b) *b*-values and c) apparent diffusion coefficients of sodium.

To unveil the relevant kinetic factors contributing to the enhanced performance of coated samples, cyclic voltammograms at increasing sweep rates were recorded allowing us to determine the possible effects of these distinctive features on the kinetic response (Figure 5a). For this purpose, three anodic peaks (labeled as Peaks 1–3) and their cathodic counterparts (Peaks 4–6). The analysis of their current maxima (*I*_*p*_) versus the sweep rate ν follows a linear relationship, as described by Equation 1:^50^
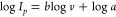
1

The *b*-values, inferred from the slope, take values from 0.5 to 1 (Figure S5, Supporting Information). Those values close to 0.5 indicate a predominant faradic diffusion-limited reaction, while those close to 1.0 are typical of a capacitive response. This capacitive behavior arises from the electrostatic interactions between the surface of the substrate and the Na^+^ incoming and outgoing Na^+^ ions. The deposition of a coating agent certainly modifies the textural properties of the metal oxide surface, thereby altering the activation barriers associated with the capacitive process and fast exchange of Na^+^.^51^ The *b*-values displayed in Figure 5b reveal a non-negligible capacitive behavior at those peaks attributable to the nickel reaction, which is responsible for the largest contribution to the overall charge and discharge capacity. However, the four-volt plateau exhibits a predominant faradic behavior. Regarding the effect of NaNbO_3_ content, larger values were determined for samples with the highest amount of the coating agent.

Cyclic voltammograms can also be an interesting source of data to calculate the apparent diffusion coefficients (*D*_*Na*^+^_^*app*^). Randles-Sevcik established a linear relationship between the current intensity at the peak maxima (*I*_*p*_) and the square root of the sweep rate, according to Equation 2:^52,53^
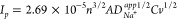
2

This equation is expressed in terms of the number of electrons transferred in the redox reaction (*n*), the electrode area (*A*), and the bulk concentration of electroactive Na^+^ (*C*). From the slopes of the linear plots shown in Figure S6 (Supporting Information), the sodium diffusion coefficients (*D*_*Na*^+^_^*app*^) were determined and depicted in Figure 5c. These coefficients took maxima values for peaks 2 and 5, while lesser values occur close to the upper and lower limit voltages. Commonly, the absence of vacancies or Na^+^ at the beginning and the end of the insertion process, respectively, is the origin of the differences between peaks. The higher values were recorded for NMO@Nb2 and NMO@Nb3, while the lowest values were determined for the bare and NMO@Nb1 samples. To summarize, the high capacitive contribution and apparent diffusion coefficients promote a fast Na^+^ exchange during cell cycling for NMO@Nb2 and NMO@Nb3 even at the highest studied rate.

**Figure 6:**
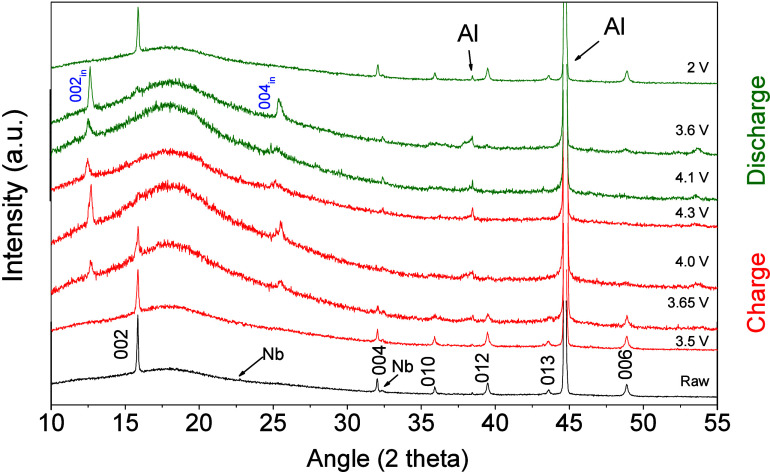
Ex-situ XRD patterns of NMO@Nb3 electrodes partially charged and subsequently discharged. Reflections coming from the Al support are marked as Al. In black, the *hkl* Miller indexes corresponding to the P2-type structure. In blue, new reflections ascribable to solvent co-intercalation.

Focusing on the best-performing sample. Figure 6 shows ex-situ XRD patterns recorded for the NMO@Nb3 sample partially charged and subsequently discharged. Charging at 3.65 V the P2-type structure coexists with a new phase, characterized by new peaks at ca. 12.5° and 25° 2θ. On charging over 4 V, these new peaks replace the reflections ascribable to the P2-type structure. These peaks are characteristic of a cointercalated phase in which solvent molecules appear within the sodium slabs,^54^ characterized by a spacing close to 14 Å (Table S1). Further discharge involved the retrieval of the P2 phase. Previous reports have revealed that the combined contribution of both the reversible electrolyte cointercalation and peroxo oxidation favor a fast-kinetics electrode behavior.^55^

**Figure 7:**
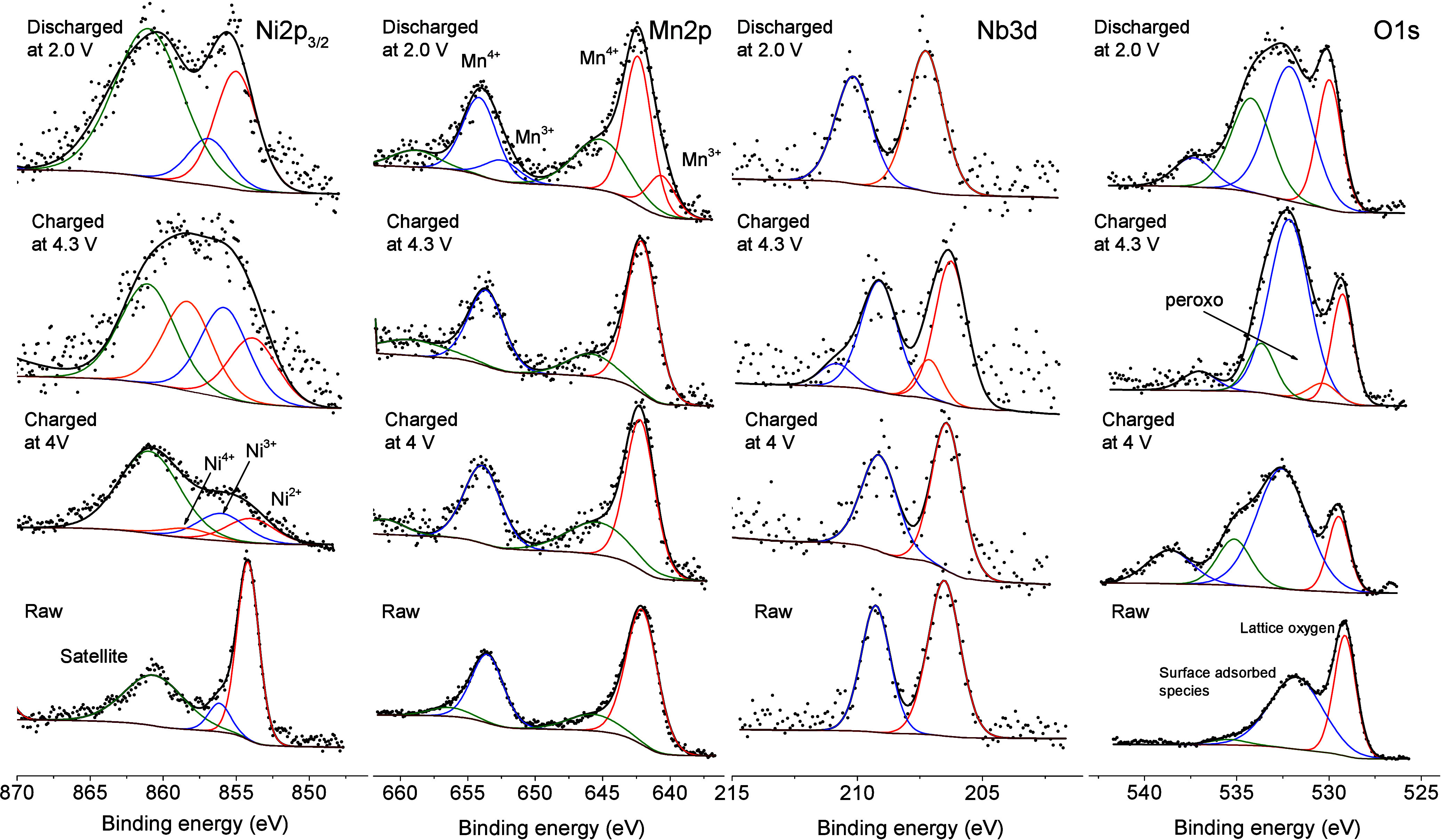
XPS spectra at the Ni 2p_3/2_, Mn 2p, Nb 3d, and O 1s core levels recorded for raw NMO@Nb3 and selected charged and discharged electrodes.

The chemical state of elements at the particle surface was determined by X-ray photoelectron spectroscopy. Figure S4 shows the survey spectrum evidencing the presence of the expected elements, besides the contribution of Na^+^. The absence of a signal splitting in the Na 1s subspectrum indicates that a significant difference in iconicity is undetectable between Na^+^ belonging to either the layered oxide or the NaNbO_3_. Figure 7 shows the Ni 2p_3/2_, Mn 2p, Nb 3d, and O 1s core level spectra. The Ni 2p_3/2_ profile for the raw material is composed of a band at 854.2 eV with a small shoulder at 856.1 eV respectively attributed to the presence of Ni^2+^ and Ni^3+^. The presence of the latter valency should be associated with oxidative processes affecting particle surface.^56^ The Mn 2p profile features asymmetric Mn 2p_3/2_ and Mn 2p_1/2_ bands respectively located at 641.9 and 653.5 eV and their corresponding satellites, being ascribable to the presence of tetravalent manganese.^57^ Finally, Figure 7 shows the O 1s spectrum in which the presence of a large peak at 529.1 eV shreds of evidence lattice oxygen, while those oxygen atoms associated with surface adsorbed species appear at 531.8 and 535.5 eV.^58^ Otherwise, the Nb 3d subspectrum features two main bands at 206.5 eV (Nb 3d_5/2_) and 209.3 eV (Nb 3d_3/2_) attributed to the presence of pentavalent vanadium.^59^

The XPS spectra of charged and discharged electrodes recorded at the Ni 2p3/2 core level evidenced the participation of the Ni^4+^/Ni^3+^/Ni^2+^ oxidation states along the electrochemical oxidation and further reversibility on discharge (Figure 7). The observation of the Mn 2p subspectra did not show appreciable changes during charge, as expected from the unavailability of higher oxidation states to Mn. Further discharge unveiled a weak shoulder to low energy bindings belonging to minor amounts of Mn^3+^, as mentioned above. The changes at the O 1s core level spectra are mostly attributable to an increase of the surface adsorbed species, coming from the formation of a surface film. Otherwise, a small band at 530.3 eV can be deconvoluted in the spectrum recorded for the fully charged electrode, which commonly relates to the formation of peroxo species.^49^ This band further disappeared in the fully discharged electrode, evidencing the reversibility of the oxo/peroxo redox couple. Finally, the Nb 3d subspectra revealed the appearance of a new band of Nb 3d shifted to a higher energy binding after full charge, which eventually became predominant after full discharge. The possibility of assigning this phenomenon to a niobium oxidation is not feasible, as will be mentioned below. Otherwise, a change in ionicity may explain this observation. The detrimental presence of HF traces in the electrolyte can be efficiently scavenged according to Equation 3:
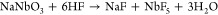
3This transformation in metal fluorides significantly diminishes the acidity of the electrolyte, thereby postponing the onset of cathode corrosion during cycling.^60−62^

**Figure 8:**
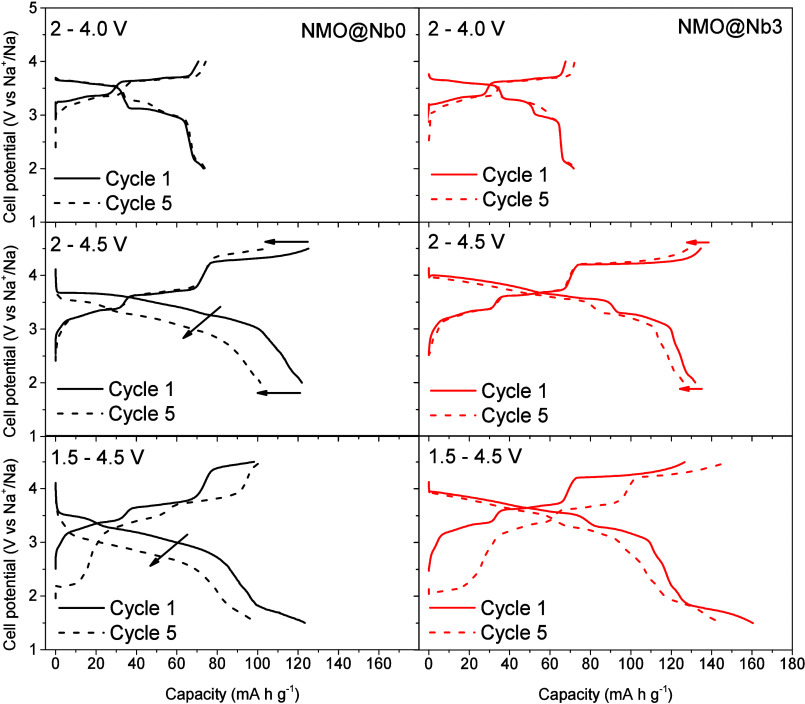
Galvanostatic charge and discharge curves of sodium half-cells assembled with NMO@Nb0 and NMO@Nb3 recorded at 2–4.0 V, 2–4.5 V, and 1.5–4.5 V at C/10.

The contribution of the four-volt plateau to the capacity retention was untangled by recording several cycles at different potential windows for the NMO@Nb0 and NMO@Nb0 (Figure 8). While noticeable differences were not observed when the upper cutoff voltage was set at 4.0 V, the increase of the upper limit to 4.5 V evidenced the good reversibility of this plateau for NMO@Nb3 as compared to the bare sample. Thus, a high Coulombic efficiency of this high voltage region can be also considered a beneficial factor to the cyclability of the coated sample.

**Figure 9:**
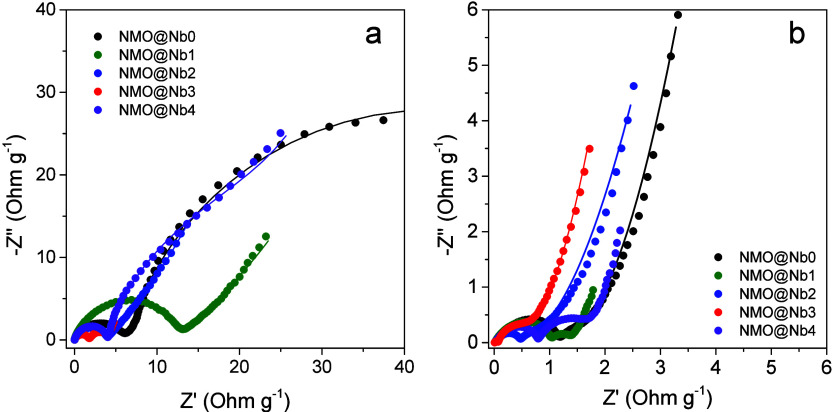
Nyquist plots of the studied samples recorded after a) the 1st cycle at C/10 and b) the 100th cycle at 1C.

Because the NaNbO_3_ coating involves significant changes at the particle surface, impedance spectra were recorded to unveil the influence of the resistance at the electrode–electrolyte interphase. Nyquist plots recorded after the first cycle at C/10 are depicted in Figure 9a. The semicircles at high and medium frequencies are currently related to capacitive and resistive phenomena occurring at the surface layer and charge-transfer reaction at the interphase. Figure S7 shows the equivalent circuit used to fit these plots and calculate the cell resistance at the surface layer (R_sl_) and charge-transfer reaction (R_ct_) (Table 3). Comparatively negligible values were recorded for the ohmic drop at the electrolyte (R_el_) and they will not be here discussed. Low impedance values were determined for NMO@Nb2 and NMO@Nb3, which may explain their better performance at the highest kinetic rate. Nyquist plots were also recorded after the 100th cycle at 1C. Figure The overall impedance decreased as a result of the progressive stabilization of the interphase during cycling to sodium migration. The most relevant result is the low R_sl_ value for NMO@Nb3 which evidence the capability of the Nb-containing layer to exert its protective effect while preserving the sodium migration through the interphase.

**Table 3:** Resistance Values from the Impedance Spectra of NaNbO_3_-coated P2-Na_2/3_Ni_1/3_Mn_2/3_O_2_ Electrodes after 1 Cycle at Different C/10 and the 100th cycle at 1C

	R_el_/Ohm g	R_sl_/Ohm g	R_ct_/Ohm g
After 1st cycle at C/10			
NMO@Nb0	0.007	6.276	74.020
NMO@Nb1	0.004	12.146	2.574
NMO@Nb2	0.016	1.868	1.860
NMO@Nb3	0.015	1.795	2.248
NMO@Nb4	0.017	4.064	8.029
After 100th cycle at 1C			
NMO@Nb0	0.017	1.241	0.318
NMO@Nb1	0.014	1.083	0.251
NMO@Nb2	0.011	0.552	0.251
NMO@Nb3	0.010	0.070	0.500
NMO@Nb4	0.013	0.764	0.814

**Figure 10:**
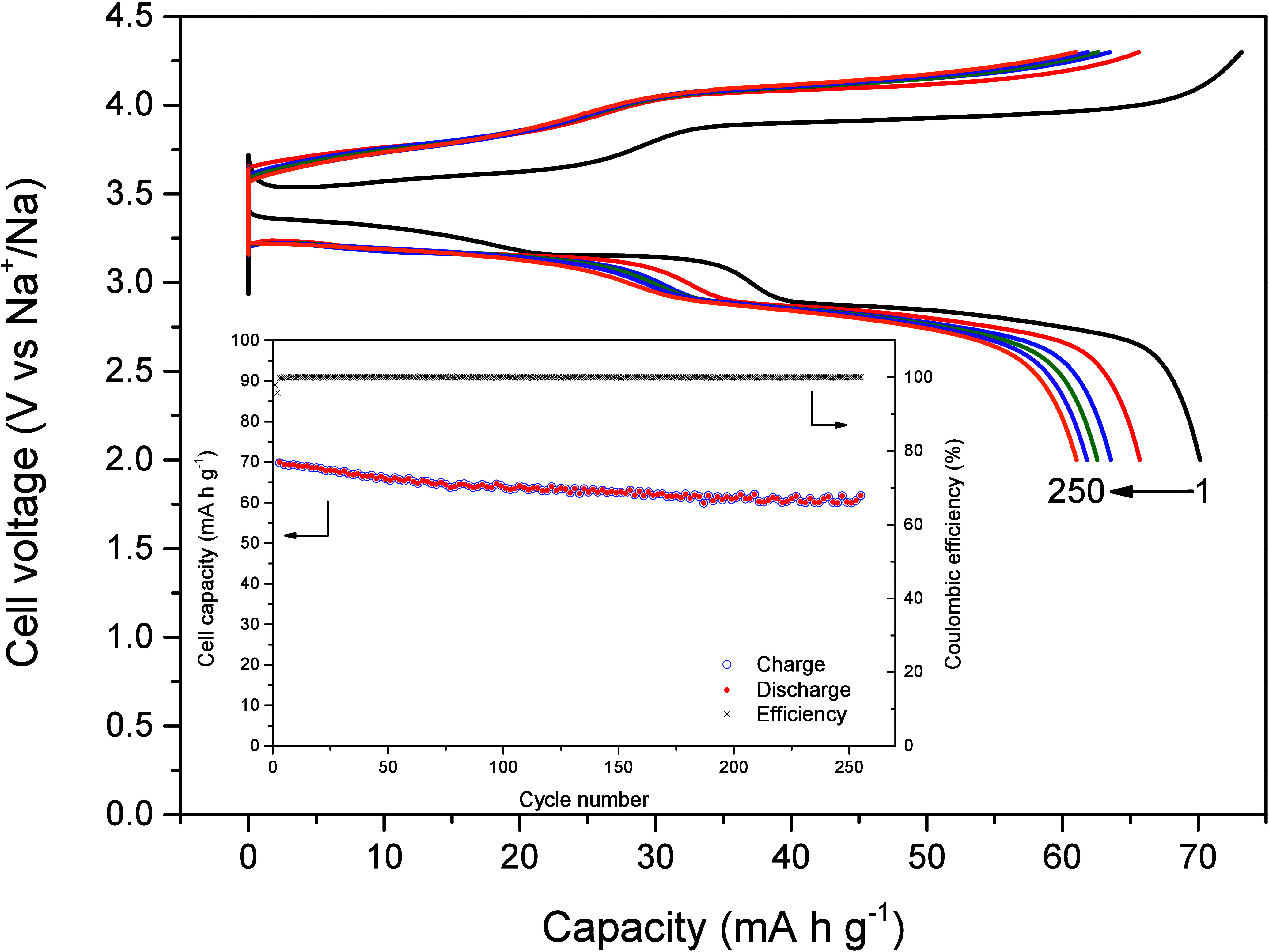
Extended galvanostatic cycling at −15 °C of a sodium half-cell assembled with NMO@Nb3 under a 1C rate.

Future batteries pose a challenge for widespread use in cold climates due to difficulties ensuring reliable operation below 0 °C. Slower electrochemical reactions at these temperatures lead to capacity loss and, last, cell failure.^63^ Encouraged by the promising electrochemical performance of NMO@Nb3, we investigated its low-temperature cycling performance at −15 °C. Figure 10 shows the capacity retention of the latter sample when cycled at 1C. The sodium half-cell delivered an initial discharge capacity of 70 mA h g^–1^ that decreased to 61 mA h g^–1^ exhibiting a good Coulombic efficiency.

## 4 Conclusions

This study demonstrates the effectiveness of NaNbO_3_ as a coating agent for P2–Na_2/3_Ni_1/3_Mn_2/3_O_2_ cathodes in sodium-ion batteries. The coating significantly improves both kinetic behavior and cyclability, as evidenced by electrochemical measurements. XRD analysis reveals no Nb incorporation into the cathode structure, suggesting a physical interaction between the coating and the cathode material. XRF analysis confirmed the expected chemical composition of the coated samples, while EDX mapping evidence the uniform dispersion of elements in the sample. Electron micrographs revealed the attachment of the coating particles to the surface of the layered particles of Na_2/3_Ni_1/3_Mn_2/3_O_2_. XPS spectra, taken at various points during the first cycle, indicated that the Ni^4+^/Ni^3+^ and Ni^3+^/Ni^2+^ redox pairs, along with the partially reversible oxidation of oxide to peroxide anions, were the primary contributors to cell capacity. Ex-situ XRD patterns evidenced the appearance of a reversible co-intercalated phase. Both the formation of the latter phase and peroxo anions are favorable factors inducing a fast kinetic. The analysis of cyclic voltammograms recorded at escalating sweep rates, both at the start and end of cycling revealed the kinetic attributes of the electrode materials under study. Samples coated with 2–3% NaNbO_3_ demonstrated superior rate capability, as evidenced by their high capacitive contribution and apparent diffusion coefficients recorded after the first cycle at C/10. Their low impedance at the electrode–electrolyte interface undoubtedly aids in delivering the highest capacity at 5C. Further cycling at 1C revealed improved cyclability in the bare and 3% coated samples, which exhibited a superior behavior, potentially due to their higher diffusion coefficients on charging. Lastly, the excellent cyclability below 0 °C of the sample coated with 3% NaNbO_3_ suggests that this material could be a promising cathode for sodium-ion batteries.

**Scheme 1:**
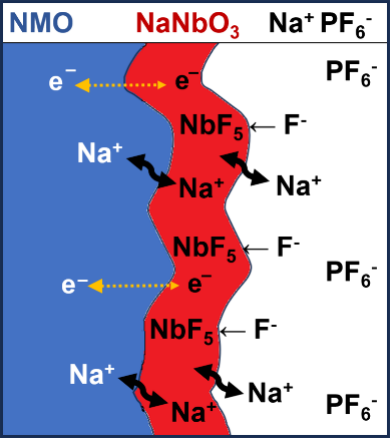
Schematic Representation of Sodium-Ion and Electron Transport through the NMO-NaNbO_3_-Electrolyte Interphases and Fluoride Scavenging Process

As reported in the suggested reference for Li_x_NbO_y_, due to the strong Nb–O bond, the Nb-rich surface can form an electrochemically active protective layer against electrolyte corrosion and deleterious phase transition.^64^ This together with the fluoride scavenging process cause the enhancement in the electrode material’s performance (new Scheme 1). However, due to the poor electronic conductivity of the coating^65^ and its nonelectroactivity, large amounts are detrimental to the electrochemical performance (Scheme 1). The composition that represents a compromise of these antagonist effects is 3% NaNbO_3_.

## Supporting Information Available

Calculated unit cell parameters of the samples, XRD pattern of NaNbO_3_, EDX spectrum and maps of elements, galvanostatic curves for NaNbO_3_ in Na cells, XPS survey spectrum, and Na 1s subspectrum, logarithmic current-scan rate plots, peak current versus the square root of scan rate plots, equivalent circuits used for EIS, and performance comparison table with literature reports (PDF)
