# Supplementary material for: Improving the Performance of the Layered Nickel Manganese Oxide Cathode of Sodium-Ion Batteries by Direct Coating with Sodium Niobium Oxide
Source: ACS Appl Mater Interfaces. 2024 Oct 9;16(42):56975–86. doi: 10.1021/acsami.4c09706 (PMC11503625; doi:10.1021/acsami.4c09706)
Supplement: Supplementary file 1 — am4c09706_si_001.pdf [file am4c09706_si_001.pdf]

Supporting Information

Improving the performance of the layered nickel manganese oxide cathode of sodium-ion batteries by direct coating with sodium niobium oxide

Sergio Lavela [b], Antônio Carlos do Nascimento Santos [a], Fabiana Villela da Motta [a], Mauricio Roberto Delmonte Bomio [a], Pedro Lavela [b], Carlos Pérez Vicente [b], José Luis Tirado [b,*]

[a] LSQM – Laboratory of Chemical Synthesis of Materials – Department of Materials Engineering, Federal University of Rio Grande do Norte – UFRN, P.O. Box 1524, Natal, RN, (Brazil)

[b] Departamento de Química Inorgánica e Ingeniería Química. Instituto Químico para la Energía y el Medioambiente. Universidad de Córdoba. Edificio Marie Curie. Campus de Rabanales 14071 Córdoba (Spain).

*Corresponding author
Tel..: +34 957 218 637
e-mail address: iq1ticoj@uco.es

Table S1. Cell parameters of the NMO@Nb3 electrodes partially charged and subsequently discharged, corresponding to the DRX patterns included in Fig. 6.

Sample		Cell parameters		
		a / Å (a)	P2, c / Å	Co-intercalated, c / Å
Raw electrode		2.882 (4)	11.163 (8)	---
Charge	3.5 V	2.885 (1)	11.157 (4)	---
	3.65 V	2.881 (3)	11.12 (1)	13.87 (2)
	4.0 V	2.881 (b)	---	13.88 (2)
	4.3 V	2.881 (b)	---	14.06 (2)
Discharge	4.1 V	2.881 (b)	---	14.00 (3)
	3.6 V	2.881 (b)	11.13 (2)	13.87 (1)
	2.0 V	2.885 (2)	11.149 (9)	---

(a) Constrained, $a_{P2} = a_{intercalated}$

(b) not refined

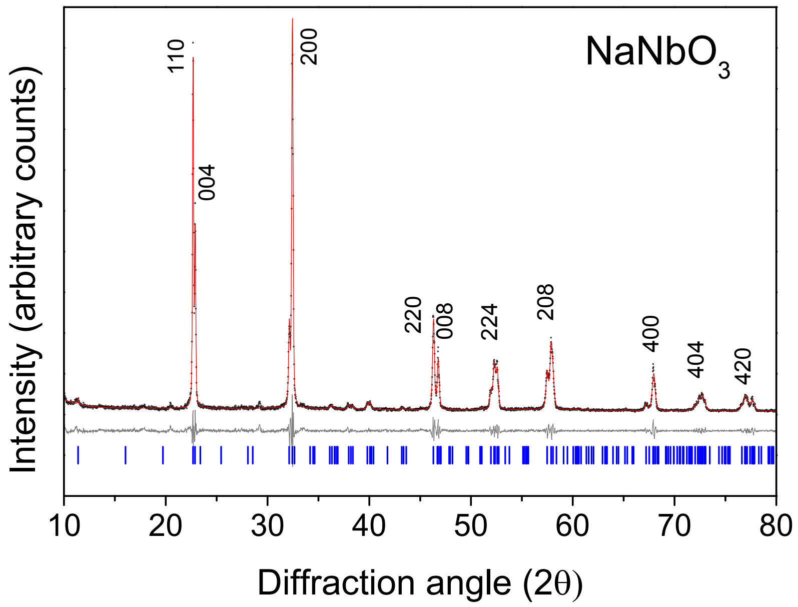

Figure S1. X-ray diffraction patterns of $NaNbO_3$ used as coating agent (black dots). Calculated pattern (red), and differential curves (grey) are included. DIF patterns for $NaNbO_3$ (PDF card No. 33-1270) (blue) is depicted.

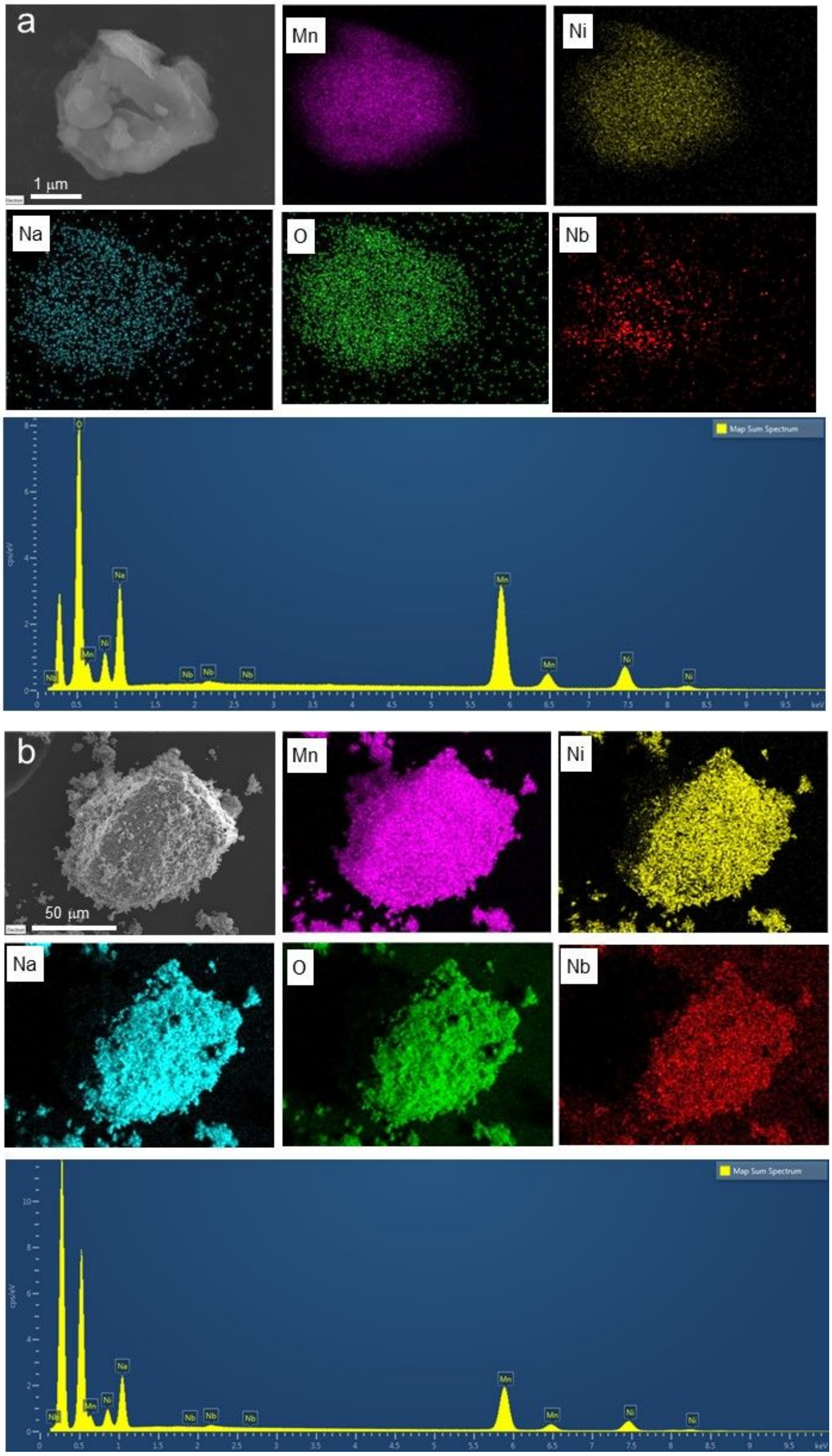
a
1 μm
Mn
Ni
Na
O
Nb
Map Sum Spectrum
b
50 μm
Mn
Ni
Na
O
Nb
Map Sum Spectrum

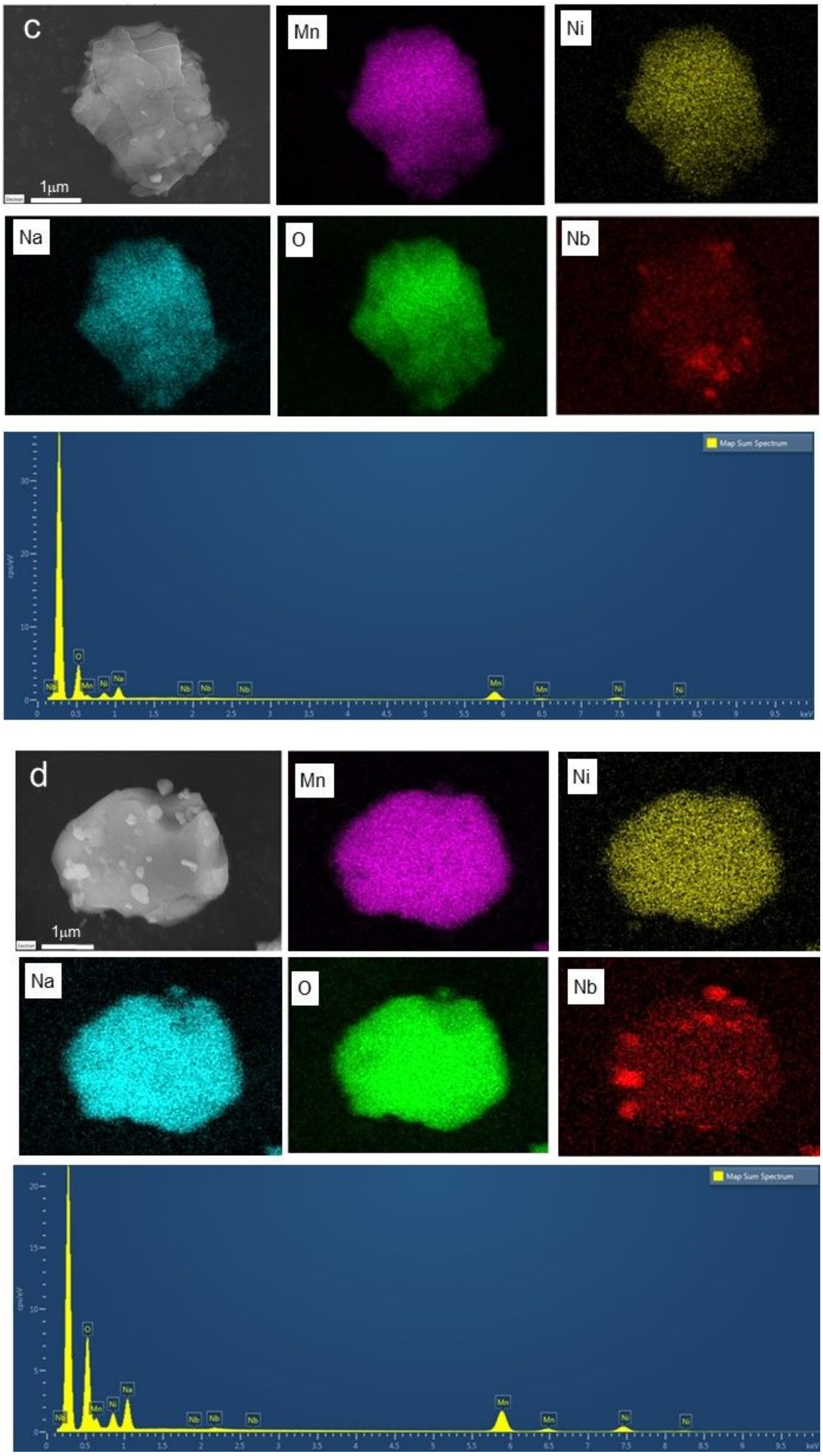

Figure S2: EDX spectrum and maps of elements Mn. Ni, Na, O and Nb for a) NMO@Nb1; b) NMO@Nb2; c) NMO@Nb3 and d) NMO@Nb4.

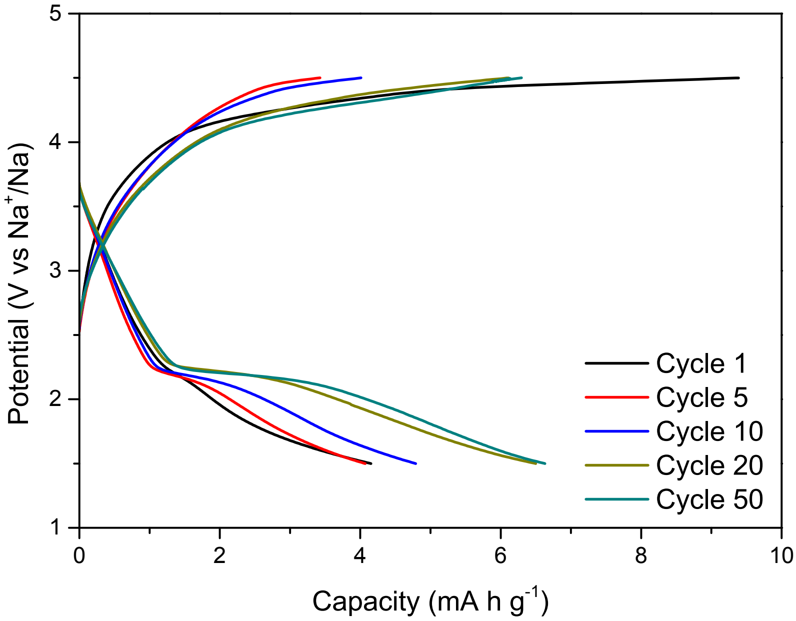

Figure S3. Galvanostatic charge and discharge curves of sodium half-cells assembled with pure $NaNbO_3$ coating agent, recorded between 1.5 and 4.5 V at C/10.

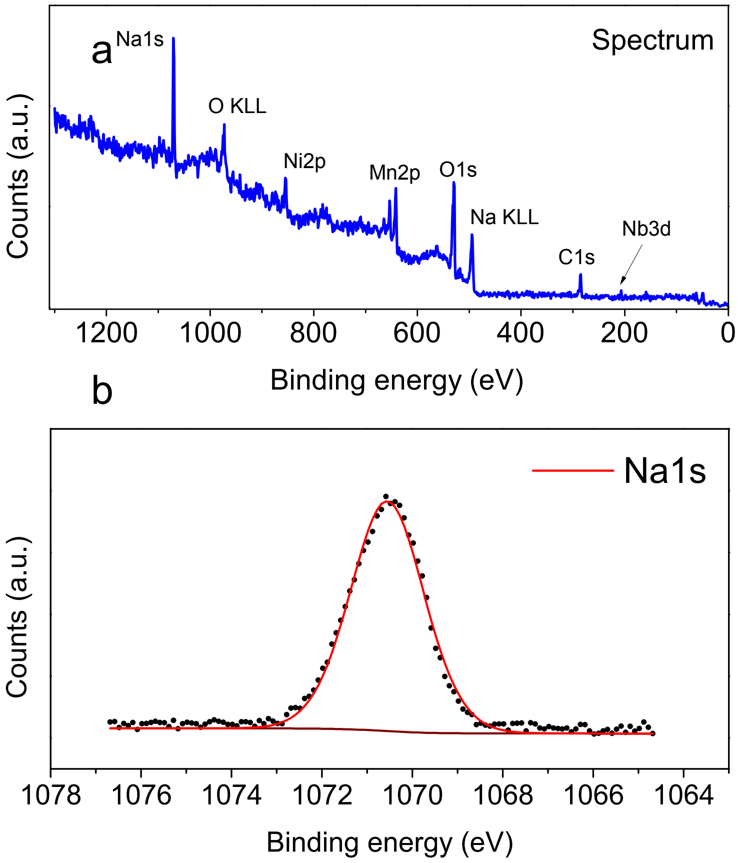

Figure S4. a) XPS survey spectrum and b) sub-spectrum at the Na1s core level recorded for raw NMO@Nb3.

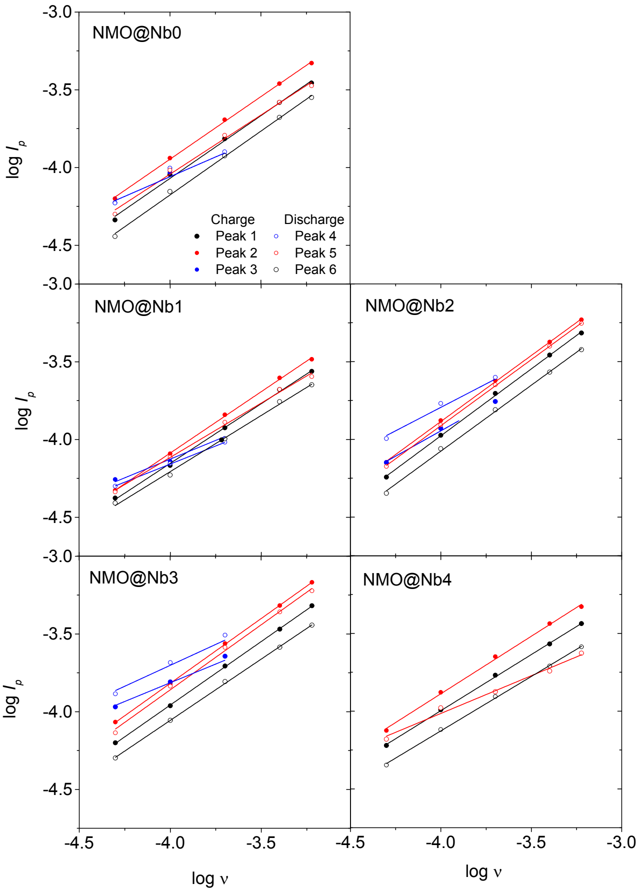

Figure S5: Logarithmic Current-scan rate plots for anodic (1-3) and cathodic (4-6) peaks in cyclic voltammograms (Figure 5a) of bare and coated samples. Cells were pre-activated with one cycle at C/10. Linear fits are shown as overlapping lines.

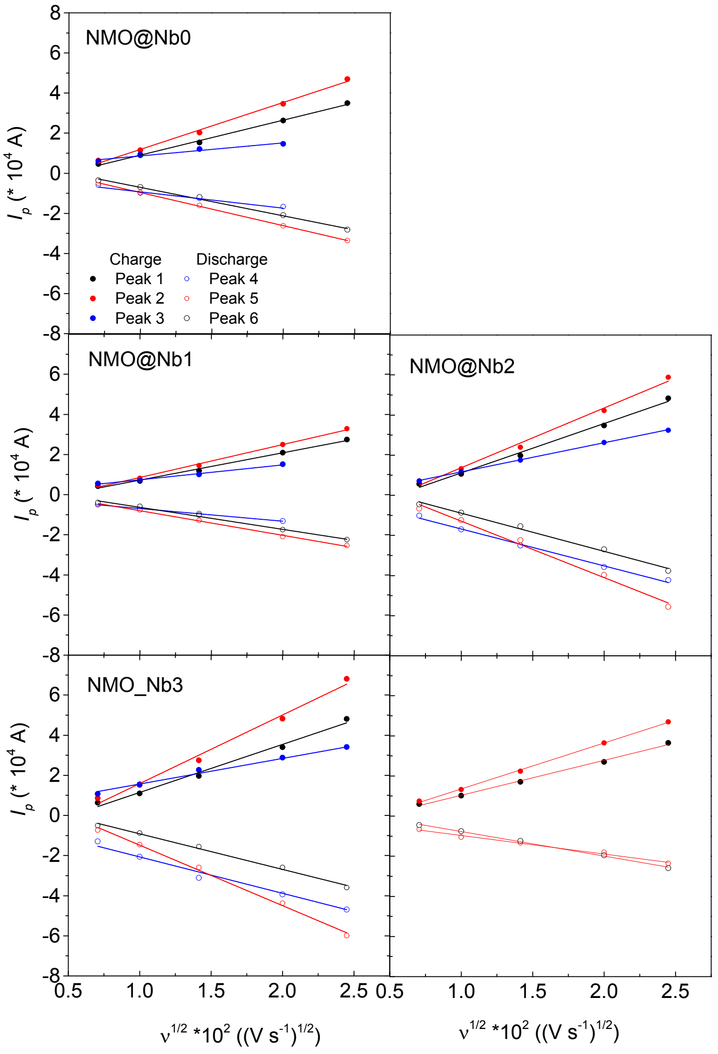

Figure S6: Plots of the peak current (I_p) versus the square root of scan rate ($v^{1/2}$) for anodic (1-3) and cathodic (4-6) peaks for the studied samples after one cycle at C/10. Linear fits are overlaid on the data points (symbols). These values are extracted from the cyclic voltammograms pictured in Figure 5a.

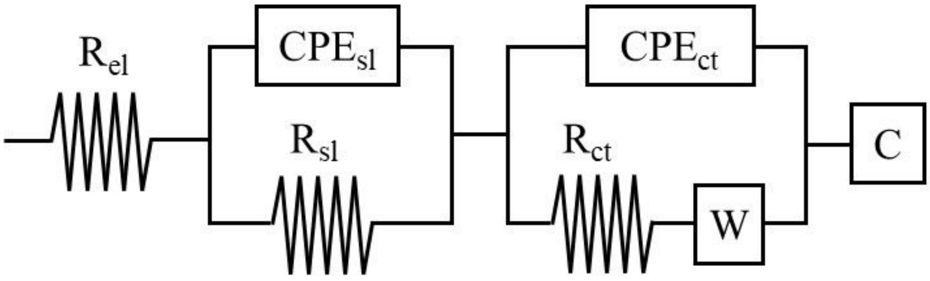

Figura S7: Equivalent circuits used for the fitting of the impedance spectra of bate and coated samples after the first cycle at C/10 and 100th at 1C.

Table S2: Comparison of previously reported electrochemical performances of related cathodes with our best material.

	Low rate mA h g^{-1} (C rate or mA g^{-1}) voltage limits /V	High rate mA h g^{-1} (C rate or mA g^{-1}) voltage limits /V	Reference
$Na_{0.67}Fe_{0.4}Mn_{0.5}Mg_{0.1}O_2$	127.5 (C/2) 2.0-4.3	ca. 50, (5C) 2.0-4.3	[1]
$Na_{0.67}Ni_{0.23}Mg_{0.1}Mn_{0.67}O_2$	105 (48) 2.0-4.5	74 (384) 2.0-4.5	[2]
$Na_{2/3}Ni_{2/9}Mg_{1/9}Mn_{2/3}O_2$	117.1 (10) 2.0-4.5	--	[3]
CuO-coated $Na_{2/3}[Ni_{1/3}Mn_{2/3}]O_2$	101 (C/10) 2.5-4.3	45 (5C) 2.5-4.3	[4]
$Na_{2/3}[Ni_{1/3}Mn_{2/3}]O_2$	87 (C/10) 2.3-4.1	62.4 (2C) 2.3-4.1	[5]
NMO@Nb3	122 (C/10, 18) 2.0-4.3	48 (5C, 900) 2-4.3<br>71 (2C, 360) 2-4.3	This work

[1] J. Wang, J. Luo, B. Zhao, G. Zhao, B. Huang, Structural stability of P2-$Na_{0.67}Fe_{0.5}Mn_{0.5}O_2$ by Mg doping, Ionics 29 (2023) 833–842. https://doi.org/10.1007/s11581-022-04845-4.

[2] H. Hou, B. Gan, Y. Gong, N. Chen, C. Sun, P2-Type $Na_{0,67}Ni_{0.23}Mg_{0.1}Mn_{0.67}O_2$ as a High-Performance Cathode for a Sodium-Ion Battery, Inorg. Chem. 55 (2016) 9033−9037. https://doi.org/10.1021/acs.inorgchem.6b01515.

[3] W. Zhao, H. Kirie, A. Tanaka, M. Unno, S. Yamamoto, H. Noguchi, Synthesis of metal ion substituted P2-$Na_{2/3}Ni_{1/3}Mn_{2/3}O_2$ cathode material with enhanced performance for Na ion batteries, Mater. Lett. 135 (2014) 131–134. http://dx.doi.org/10.1016/j.matlet.2014.07.153.

[4] R. Dang, Q. Li, M. Chen, Z. Hu, X. Xiao, CuO-Coated and Cu^{2+}-doped Co-modified P2-type $Na_{2/3}[Ni_{1/3}Mn_{2/3}]O_2$ for sodium-ion batteries, Phys. Chem. Chem. Phys. 21 (2019) 314–321. https://doi.org/10.1039/c8cp06248j.

[5] D.H. Lee, J. Xu, Y.S. Meng, An advanced cathode for Na-ion batteries with high rate and excellent structural stability, Phys. Chem. Chem. Phys. 15 (2013) 3304. https://doi.org/10.1039/c2cp44467d.
